# Clinical features of progressive supranuclear palsy

**DOI:** 10.3389/fnagi.2023.1229491

**Published:** 2023-08-30

**Authors:** Yafei Wen, Qijie Yang, Bin Jiao, Weiwei Zhang, Jingyi Lin, Yuan Zhu, Qian Xu, Hui Zhou, Ling Weng, Xinxin Liao, Yafang Zhou, Junling Wang, Jifeng Guo, Xinxiang Yan, Hong Jiang, Beisha Tang, Lu Shen

**Affiliations:** ^1^Department of Neurology, Xiangya Hospital, Central South University, Changsha, China; ^2^National Clinical Research Center for Geriatric Disorders, Central South University, Changsha, China; ^3^Engineering Research Center of Hunan Province in Cognitive Impairment Disorders, Central South University, Changsha, China; ^4^Hunan International Scientific and Technological Cooperation Base of Neurodegenerative and Neurogenetic Diseases, Changsha, China; ^5^Key Laboratory of Hunan Province in Neurodegenerative Disorders, Central South University, Changsha, China; ^6^Department of Radiology, Xiangya Hospital, Central South University, Changsha, China; ^7^Department of Geriatrics Neurology, Xiangya Hospital, Central South University, Changsha, China; ^8^Key Laboratory of Organ Injury, Aging and Regenerative Medicine of Hunan Province, Changsha, China

**Keywords:** progressive supranuclear palsy, phenotype, Richardson’s syndrome, parkinsonism, longitudinal MRI

## Abstract

**Background:**

Progressive supranuclear palsy (PSP) is a clinically heterogenous atypical parkinsonian syndrome. Therefore, early recognition and correct diagnosis of PSP is challenging but essential. This study aims to characterize the clinical manifestations, magnetic resonance imaging (MRI), and longitudinal MRI changes of PSP in China.

**Method:**

Clinical and MRI presentations were compared among 150 cases with PSP. Then the longitudinal MRI changes among 20 patients with PSP were further explored. Additionally, a series of midbrain-based MRI parameters was compared between PSP-P and PD.

**Results:**

Throughout the course of the disease, there were differences in the symptoms of the fall and hand tremor between the PSP-RS and PSP-P. There were significant differences in the six midbrain-based MRI parameters between the PSP-RS and the PSP-P, including hummingbird sign, midbrain diameter, midbrain to pons ratio (MTPR), midbrain area, midbrain area to pons area ratio (Ma/Pa), and midbrain tegmental length (MBTegm). Longitudinal MRI studies revealed that the annual rel.ΔMTPR and rel.Δ (Ma/Pa) for PSP were 5.55 and 6.52%, respectively; additionally, PSP-RS presented a higher decline rate than PSP-P. Moreover, MTPR ≤0.56, midbrain diameter ≤ 0.92, midbrain area ≤ 1.00, and third ventricle width ≤ 0.75 could identify PSP-P from PD.

**Conclusion:**

PSP-P differs from PSP-RS regarding clinical manifestations, MRI, and longitudinal MRI changes. MRI parameters could be potential imaging markers to identify PSP-P from PD.

## 1. Introduction

Progressive supranuclear palsy (PSP) is an atypical parkinsonian syndrome associated with a specific four repeat (4R) tau neuropathology at autopsy. The classic movement disorder clinical phenotype now was referred to as Richardson’s syndrome (PSP-RS). It was first described in 1964 as a clinicopathological entity, characterized by vertical supranuclear gaze palsy, early postural instability with unprovoked falls, progressive axial rigidity, and mild dementia (Steele et al., 1964; Ling, 2016; Armstrong, 2018). Following its initial description, other phenotypes of PSP have been gradually described, known as the variant PSP (vPSP), which greatly broadened the clinical spectrum of PSP (Williams et al., 2005; Respondek et al., 2014; Owens et al., 2016; Höglinger et al., 2017). The cardinal neuropathologic features of PSP at autopsy included neurofibrillary tangles (hyperphosphorylated tau protein), along with neuronal loss and gliosis (Dickson et al., 2007). Second-generation tau tracers, such as ^[18F]^MK-6240, ^[18F]^PI-2620 and others, could be expected to be viable biomarkers for 4R tauopathies (Leuzy et al., 2019; Cassinelli Petersen et al., 2022). Tau PET imaging could enable *in vivo* visualization of PSP-related tau pathology, and play an important role in identifying PSP from atypical parkinsonisms (Jin et al., 2023).

PSP is a rare disease with an estimated prevalence of 3–6/100000 (Respondek et al., 2017); However, it is worth noting that this prevalence data mainly reflected the occurrence of PSP-RS (Stamelou et al., 2019). The true prevalence of PSP is likely underestimated due to insufficient recognition of vPSP. Among the different phenotypes, PSP-RS is the most common, followed by PSP-parkinsonism (PSP-P) (Alster et al., 2020). Although Movement Disorder Society new clinical diagnostic criteria for PSP (MDS-PSP) were published in 2017, aiming to include all PSP subtypes early, clinicians still have difficulties in diagnosing the vPSP phenotype (Höglinger et al., 2017; Ali et al., 2019).

There was a delay, usually 3 years, from the onset of symptoms to the diagnosis of PSP (Cui et al., 2020). That is halfway through the illness. PSP is a uniformly fatal disease without effective therapies, but treatment can be helpful. Moreover, potential disease-modifying therapies for PSP heighten the need for early, accurate clinical diagnosis (Boxer et al., 2017; Höglinger et al., 2017; Jabbari et al., 2020); therefore, early recognition and correct diagnosis of PSP is essential.

This study analyzed the clinical characteristics, disease progression, and MRI assessment of PSP patients in mainland China. Then, the characteristics between PSP-RS and PSP-P cases were compared. We aim to investigate the frequency of major clinical symptoms at different stages of PSP, identify easily accessible imaging parameters, and determine the predictors for early diagnosis in the clinic.

## 2. Methods

### 2.1. Study participants

A total of 150 Chinese patients with PSP were enrolled in this study. A total of 43 Chinese PD patients who had multiple visits and underwent cranial MRI were recruited. All patients underwent thorough neurological examination by two experienced neurologists at Xiangya Hospital, Central South University. The diagnoses of PD and PSP were performed according to MDS-PD and NINDS-SPSP, MDS-PSP, respectively (Litvan et al., 1996; Postuma et al., 2015; Höglinger et al., 2017). The diagnoses and clinical phenotypes were confirmed using the MDS-PSP criteria and Multiple Allocations eXtinction (MAX) rules at the last clinical follow-up evaluation (Höglinger et al., 2017; Grimm et al., 2019). The present study was approved by the Ethics Committee of Xiangya Hospital, Central South University. Written informed consent was obtained from each participant or their legal representatives.

### 2.2. Data collection

#### 2.2.1. Clinical data

Detailed information of patients with PSP was acquired. Demographic data included sex, age, age at onset (AAO), education, and family history. Data on clinical symptoms included the following seven aspects: (1) ocular symptoms, including ocular motor dysfunction, dry eyes, visual blurring, diplopia, reduced blinking rate, and apraxia of eyelid opening; (2) postural instability, including unstable walking, falls, weakness of limbs, and gait change; (3) parkinsonian symptoms, including gait freezing, bradykinesia, tremor, axial or limb rigidity, and levodopa-responsiveness; (4) cognitive impairment, including memory decline, personality and behavior change (depression/anxiety/apathy), and decreased verbal fluency; (5) bulbar dysfunction, including dysarthria and dysphagia; (6) autonomic dysfunction including dizziness, urinary frequency/urgency/urinary incontinence; and (7) other symptoms, such as sleep disorders and localized pain. Additionally, we collected all available assessment scales, including the Mini-Mental State Examination (MMSE), Montreal Cognitive Assessment (MoCA), Unified Parkinson Disease Rating Scale (motor subsection III, UPDRS III) and improvement in the acute levodopa challenge test (ALCT) parameters (Folstein et al., 1975; Nasreddine et al., 2005; Martinez-Martin and Forjaz, 2006).

#### 2.2.2. Radiographic data

All patients with PSP underwent brain MRI after symptom onset. If a patient had multiple MRI examinations of the brain, we selected the MRI image closest to the time of diagnosis. The presence of the ‘hummingbird sign’ was visually assessed as described by Kato et al. (2003). Midbrain and pons diameters, Midbrain and pons areas, midbrain tegmental length (MBTegm), cerebral peduncle angle, third ventricle width, and frontal horns width were measured as previously described (Kato et al., 2003; Oba et al., 2005; Massey et al., 2013; Eraslan et al., 2019; Picillo et al., 2020). In patients who underwent oblique coronal MRI, middle cerebellar peduncle width (MCP) and superior cerebellar peduncle width (SCP) was measured (Morelli et al., 2011; Madetko et al., 2022; Quattrone et al., 2022). All quantitative parameters were measured three times for each participant and averaged. Subsequently, midbrain to pons ratio (MTPR), midbrain area to pons area ratio (Ma/Pa), third ventricle width to frontal horns width ratio (3rdV/FH), Magnetic Resonance Parkinsonism Index (MRPI), and Magnetic Resonance Parkinsonism Index 2.0 (MRPI 2.0) were calculated. Annual reductions in MTPR and Ma/Pa and its relative changes were calculated for patients who had undergone multiple MRI examinations of the brain. Using the MTPR as an example, the formulae were defined as follows: annual ΔMTPR = (MTPR_BL_-MTPR_DI_)/(Age_DI_-Age_BL_) and annual relative ΔMTPR (rel.ΔMTPR) = (annual ΔMTPR/MTPRBL) × 100% (MTPR_BL_ refers to the baseline MTPR, and MTPR_DI_ refers to the MTPR at PSP diagnosis).

### 2.3. Statistical analyses

For all collected data, categorical variables were presented as frequency and continuous variables as mean ± standard deviation (SD). Quantitative variables were compared using the t test or the Mann Whitney U test. Qualitative variables were compared using the chi-squared test or fisher test. The frequency of symptoms and MRI parameters among different groups were compared using logistic regression model or linear regression model, adjusted by disease duration and/or age at MRI. All analyses were performed using R 4.1.1. A value of *p* <0.05 was considered statistically significant.

## 3. Results

### 3.1. Characteristics of all participants

In total, 150 patients fulfilled the clinical criteria for PSP and were enrolled in our study. Their demographic and clinical characteristics were shown in Table 1. According to the MDS-PSP criteria, 100 (66.7%) were classified as PSP-RS, 44 (29.3%) as PSP-P, and 6 (4.0%) were diagnosed with other vPSPs, including 3 cases (2.0%) of postural instability (PSP-PI), 2 cases (1.3%) of frontal lobe cognitive or behavioral presentations (PSP-F), and one (0.7%) case of corticobasal syndrome (PSP-CBS). Patients with an age at onset (AAO) between 60 and 65 years were common and accounted for 32.7% of our cohort. In the PSP cohort, the proportion of men was significantly higher than that of women (*p* = 0.003). There were no differences in AAO, MMSE, MoCA, UPDRS III, or ALCT scores between the PSP-RS and PSP-P groups; however, the disease duration in the PSP-RS group (2.57 ± 1.69 years) was significantly shorter than that in the PSP-P group (4.26 ± 2.78 years; *p < 0.001*).

**Table 1:** Demographic and clinical data of patients with PSP.

	PSP (*n* = 150)	^#^PSP-RS (*n* = 100)	^#^PSP-P (*n* = 44)	Other vPSP (*n* = 6)	^#^value of *p* (PSP-RS vs. PSP-P)
Sex (% male)	62.0	58.0	70.5	66.7	0.218
Family history (%) ^*^	10.6	9	13.6	16.7	0.587
AAO (years)	62.05 ± 7.93	62.48 ± 7.82	61.41 ± 7.51	59.67 ± 12.88	0.445
Disease duration (years)	3.21 ± 2.56	2.57 ± 1.69	4.26 ± 2.78	6.33 ± 6.77	**<0.001**
Education (years)	9.13 ± 3.17	9.16 ± 3.81	8.79 ± 3.67	11.0 ± 1.55	0.597
MMSE (score)	21.15 ± 7.03	21.05 ± 7.31	20.85 ± 6.77	24.75 ± 2.22	0.905
MoCA (score)	13.69 ± 6.48	14.16 ± 6.41	12.42 ± 6.80	–	0.335
UPDRS III (score)	38.72 ± 15.00	37.53 ± 15.98	41.21 ± 13.42	–	0.385
Improvement of ALCT	0.17 ± 0.10	0.16 ± 0.10	0.19 ± 0.10	–	0.295

PSP, progressive supranuclear palsy; PSP-RS, progressive supranuclear palsy–Richardson’s syndrome; PSP-P, progressive supranuclear palsy-parkinsonism; vPSP: variant progressive supranuclear palsy; AAO, age at onset; MMSE, mini-mental state Examination; MoCA, montreal cognitive assessment; UPDRS, unified Parkinson disease rating scale; ALCT, acute levodopa challenge test; Disease duration, the interval from symptom onset to PSP diagnosis.

* Family history refers to family members with similar symptoms to patients with PSP.

# Value of *p* refers to comparisons between the PSP-RS and PSP-P groups. *p* < 0.05 is marked in bold.

### 3.2. Initial symptoms in patients with PSP

This section described the frequency of the main initial symptom, defined as the symptom reported by the patient within 6 months of onset. At the same time, the proportions of each symptom between the PSP-RS and PSP-P groups were compared. As shown in Table 2, 62.7% of the patients with PSP had bradykinesia as the initial symptom. Ocular symptoms were important suggestive symptoms of PSP, but it was almost absent in the early stages. For PSP-RS group, bradykinesia was the most common initial symptom, followed by unstable gait and falls. For PSP-P group, the symptoms with the highest proportions at onset were bradykinesia and tremors. Compared with PSP-P, patients with PSP-RS were more likely to experience unsteady walking or falls in the early stage (both *p* = 0.01), less likely to have tremor at onset (*p* < 0.001).

**Table 2:** Initial symptoms among the chief complaints in patients with PSP.

	PSP (*n* = 150)	^#^PSP-RS (*n* = 100)	^#^PSP-P (*n* = 44)	Other vPSP (*n* = 6)	^#^value of *p* (PSP-RS vs. PSP-P)
Ocular symptoms (%)	3.3	5.0	0.0	0.0	0.995
Unstable walking (%)	25.3	32.0	9.1	33.3	**0.010**
Falls (%)	23.3	31.0	6.8	16.7	**0.010**
Weakness of limbs (%)	11.3	13.0	9.1	0.0	0.515
Gait changes	3.3	4.0	2.3	0.0	0.425
Bradykinesia (%)	62.7	60.0	70.5	50.0	0.309
Tremors (%)	19.3	9.0	40.9	33.3	**0.001**
Limb rigidity (%)	5.3	7.0	2.3	0.0	0.224
Cognitive impairment (%)	7.3	10.0	0.0	16.7	0.991
Pseudobulbar palsy (%)	7.3	10.0	2.3	0.0	0.127
Dizziness (%)	6.0	6.0	6.8	0.0	0.671

PSP, progressive supranuclear palsy; PSP-RS, progressive supranuclear palsy; Richardson’s syndrome; PSP-P, progressive supranuclear palsy-parkinsonism; vPSP: variant progressive supranuclear palsy.

# *P*-value refers to the comparisons between the PSP-RS and PSP-P groups. *p*-value was calculated by logistic regression model, adjusted by disease duration. *p* < 0.05 is marked in bold.

### 3.3. Evolution of main symptoms of PSP

**Figure 1:**
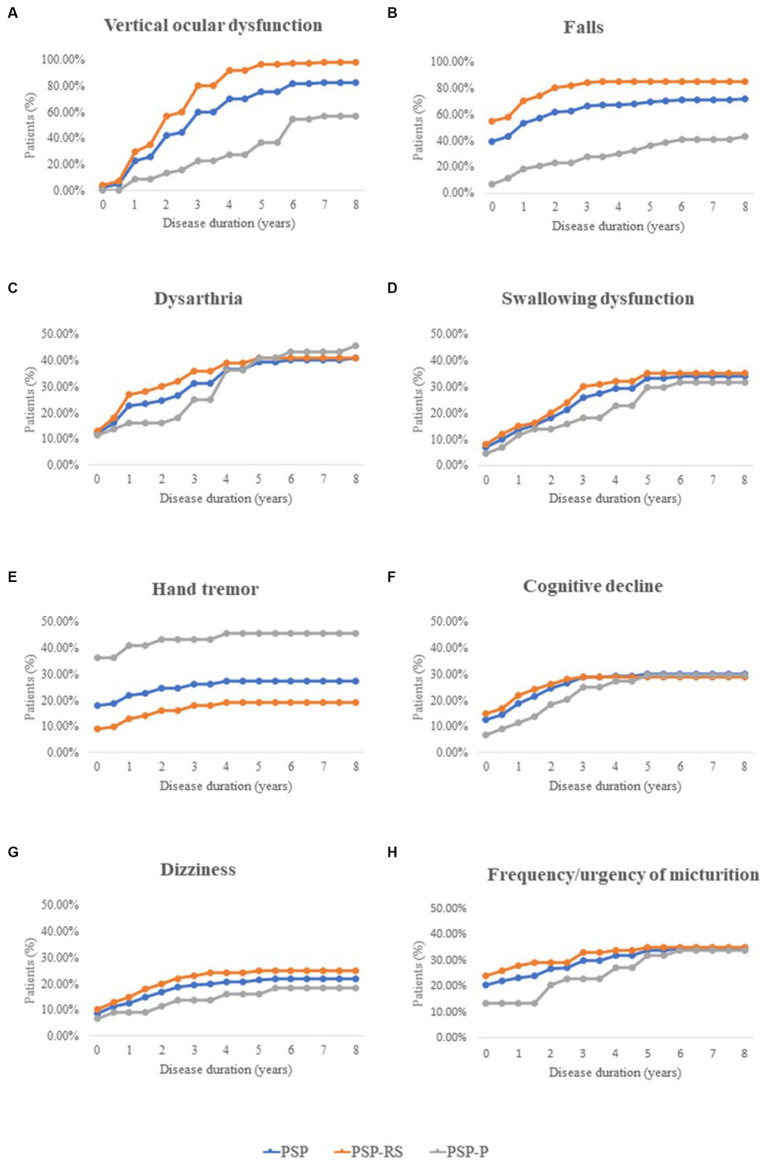
Frequency of important clinical features during different disease stages in patients with PSP, including Vertical ocular dysfunction, Falls, Dysarthria, Swallowing dysfunction, Hand tremor, Cognitive decline, Dizziness, and Frequency/urgency of micturition. PSP, progressive supranuclear palsy; PSP-RS, progressive supranuclear palsy-Richardson’s syndrome; PSP-P, progressive supranuclear palsy-parkinsonism.

Eight important and easily noticeable symptoms were selected, and the frequencies of each symptom at different stages of PSP were then summarized (Figure 1). In the PSP cohort (blue lines), the number of patients with vertical ocular dysfunction increased most rapidly as the disease progressed. Additionally, the number of patients with each selected symptom remained nearly unchanged after the sixth year of disease.

Throughout the course of the disease, the number of patients with falls was consistently highest in the PSP-RS group (orange lines), while hand tremors remained more common in the PSP-P group (gray lines).

Moreover, the number of patients with hand tremors and falls stabilized after the fourth year of disease.

The progression of pseudobulbar palsy (including dysarthria and swallowing dysfunction) and other nonmotor symptoms (including cognitive decline, dizziness, and urinary dysfunction) was similar in patients with PSP-RS and PSP-P, especially in the late stage of the disease.

#### 3.3.1. MRI parameters in patients with PSP

A total of 141 patient imaging data were available for analysis. Among them, 47.5% of patients (67/141) showed hummingbird signs. The mean midbrain diameter and area in PSP cohort were 7.88 ± 1.23 mm and 82.18 ± 20.98 mm^2^, respectively. The midbrain diameter, midbrain area, MBtegm, MTPR, and Ma/Pa of the PSP-RS group were significantly lower than those of the PSP-P group (all *p* < 0.001), while the frequency of hummingbird signs was higher in the PSP-RS group (*p* = 0.04) (Table 3). There was no significant difference in other MRI parameters between PSP-RS group and PSP-P group.

**Table 3:** MRI assessments of patients with PSP.

	PSP (*n* = 141)	^#^PSP-RS (*n* = 92)	^#^PSP-P (*n* = 43)	Other vPSP (*n* = 6)	^#^*p*-value (PSP-RS vs. PSP-P)
Hummingbird sign (%)	47.5	51.1	41.9	33.3	**0.04** ^**a**^
Midbrain diameter (mm)	7.88 ± 1.23	7.60 ± 1.08	8.43 ± 1.30	8.15 ± 1.80	**<0.001**
Pons diameter (mm)	17.13 ± 1.08	16.99 ± 1.00	17.31 ± 1.17	17.87 ± 1.22	0.26
MTPR	0.46 ± 0.07	0.45 ± 0.07	0.49 ± 0.07	0.46 ± 0.10	**0.001**
Midbrain area (mm^2^)	82.18 ± 20.98	78.32 ± 17.02	89.91 ± 25.81	85.37 ± 25.00	**0.001**
Pons area (mm^2^)	495.86 ± 53.06	489.10 ± 50.10	503.53 ± 56.65	495.86 ± 53.06	0.11
Ma/Pa	0.17 ± 0.04	0.16 ± 0.03	0.18 ± 0.04	0.16 ± 0.04	**0.006**
MRPI^##^	16.68 ± 5.76	17.37 ± 6.03	13.52 ± 3.37	–	0.27
MBTegm (cm)	9.62 ± 1.59	9.30 ± 1.32	10.30 ± 1.93	9.50 ± 1.22	**<0.001**
CPA (°)	69.60 ± 7.87	69.48 ± 8.19	69.58 ± 7.71	71.58 ± 3.13	0.90
Third ventricle width (mm)	8.67 ± 2.31	8.79 ± 2.46	8.38 ± 1.88	9.04 ± 2.91	0.16
Frontal horns width (mm)	35.57 ± 4.17	35.33 ± 4.43	36.20 ± 3.78	34.73 ± 2.08	0.61
3rdV/FH	0.24 ± 0.06	0.25 ± 0.06	0.23 ± 0.05	0.26 ± 0.08	0.08
MRPI2.0^##^	3.97 ± 1.73	4.22 ± 1.77	3.19 ± 1.60	–	0.30

PSP, progressive supranuclear palsy; PSP-RS, progressive supranuclear palsy; Richardson’s syndrome; PSP-P, progressive supranuclear palsy-parkinsonism; vPSP: variant progressive supranuclear palsy.

# *p*-value refers to the comparisons between the PSP-RS and PSP-P groups.

a *p*-value was calculated by logistic regression model, adjusted by disease duration and age at MRI. Other *p*-value was calculated by linear regression model, adjusted by disease duration and age at MRI.

## A total of 29 patients (22 in the PSP-RS group and 7 in the PSP-P group) had measurable MRPI and MRPI2.0. *p* < 0.05 is marked in bold.

### 3.4. Longitudinal MRI changes in patients with PSP

**Figure 2:**
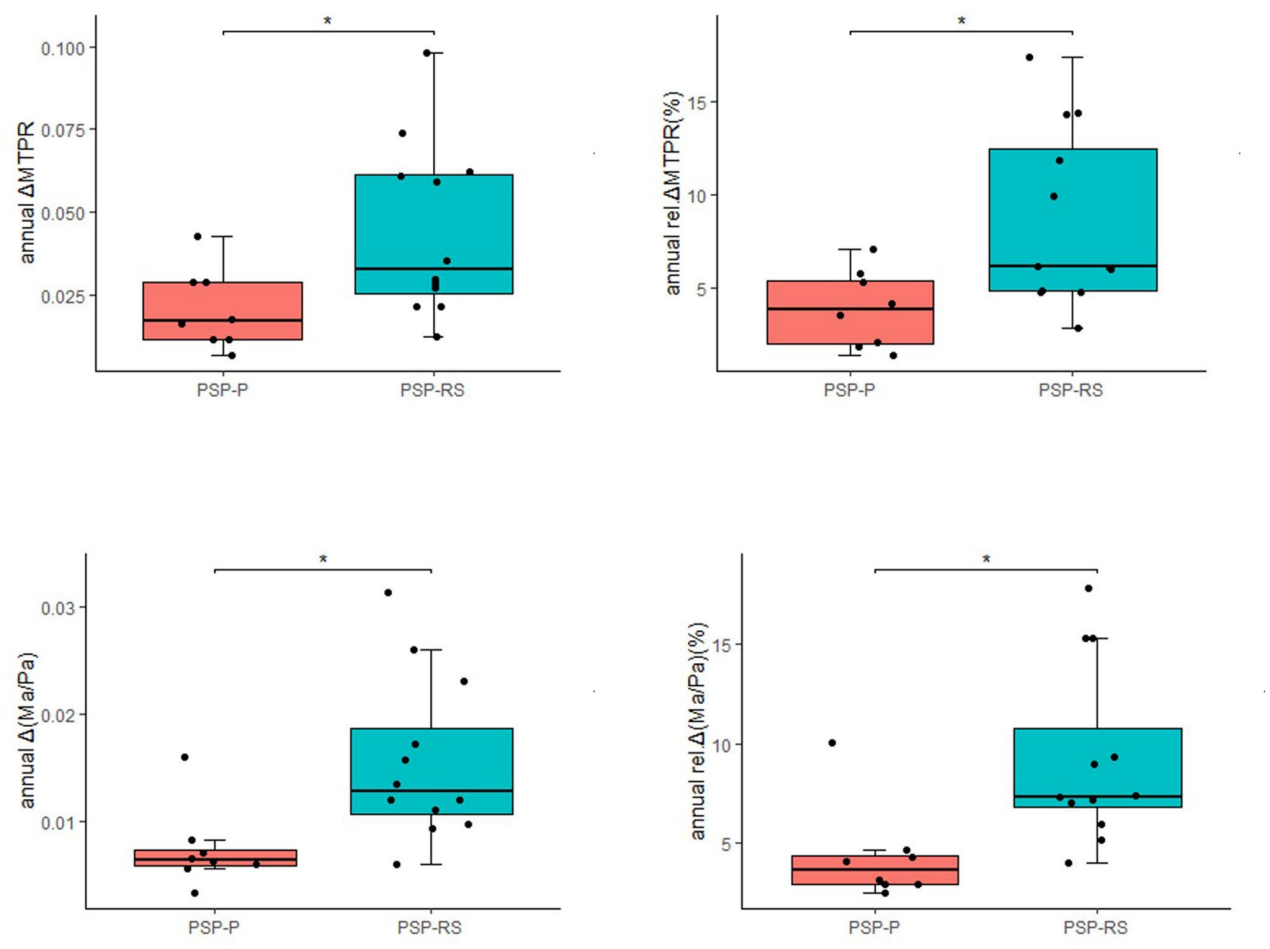
Longitudinal changes in 20 patients with PSP (*p < 0.05).

The progression of midbrain atrophy via longitudinal brain MRI was investigated. In total, twelve PSP-RS cases and eight PSP-P cases were included. The most common initial diagnoses among these 20 patients were Parkinson’s disease (PD) and parkinsonian syndrome. These patients were diagnosed with PSP approximately 2 years (range: 1.0–4.0) after the initial visit. The median annual rel.ΔMTPRs for PSP, PSP-RS, and PSP-P were 5.55, 6.16, and 3.86%, respectively, and the median annual rel.Δ (Ma/Pa) were 6.52, 7.35, and 3.62%, respectively. Compared with PSP-P, annual ΔMTPR, annual Δ (Ma/Pa), annual rel.ΔMTPRs, and annual rel.Δ (Ma/Pa) were significantly higher in PSP-RS patients (Figure 2). It was worth noting that in our cohort, significant changes in MRI were observed in two patients within half a year.

### 3.5. Demographic characteristics and MRI parameters of patients with PSP-P and PD

A total of 43 patients with PSP-P and 43 patients with PD were included in this section. The average onset age of PSP-P patients was significantly higher than PD patients (61.51 ± 7.56 vs. 55.58 ± 10.48), while the MMSE scores was significantly lower than that of PD patients (21.24 ± 6.59 vs. 26.16 ± 3.87). There was no significant difference in disease duration. Compared with those in the PD group, nine midbrain-based MRI parameters significantly decreased in the PSP-P group, including midbrain diameter, pons diameter, MTPR, midbrain area, pons area, Ma/Pa, MBtegm, third ventricle width, 3rdV/FH (all *p* < 0.05) (Table 4).

**Table 4:** Clinical information and MRI assessments of patients with PSP and patients with PD.

	PSP-P (*n* = 43)	PD (*n* = 43)	*p*-value (PSP-P vs. PSP-PD)
Sex (% male)	69.76%	69.76%	1^a^
AAO (years)	61.51 ± 7.56	55.58 ± 10.48	**0.005** ^**b**^
Disease duration (years)	4.22 ± 2.80	5.71 ± 4.81	0.083
MMSE (score)	21.24 ± 6.59	26.16 ± 3.87	**0.001** ^**b**^
Midbrain diameter (mm)	8.43 ± 1.30	10.21 ± 0.89	**<0.001**
Pons diameter (mm)	17.31 ± 1.17	16.62 ± 1.51	**0.03**
MTPR	0.49 ± 0.07	0.62 ± 0.07	**<0.001**
Midbrain area (mm^2^)	89.91 ± 25.81	116.21 ± 16.39	**<0.001**
Pons area (mm^2^)	503.53 ± 56.65	551.12 ± 55.07	**<0.001**
Ma/Pa	0.18 ± 0.04	0.21 ± 0.03	**<0.001**
MBTegm (cm)	10.30 ± 1.93	11.58 ± 0.82	**<0.001**
CPA (°)	69.58 ± 7.71	69.20 ± 9.03	0.73
Third ventricle width (mm)	8.38 ± 1.88	6.20 ± 1.95	**<0.001**
Frontal horns width (mm)	36.20 ± 3.78	35.02 ± 4.56	0.64
3rdV/FH	0.23 ± 0.05	0.18 ± 0.06	**<0.001**

PSP-P, progressive supranuclear palsy-Parkinsonism; PD, Parkinsonism disease.

a *p*-Value was calculated by Chi-squared test.

b *p*-Value was calculated by Mann Whitney *U* test; Other value of *p* were calculated by logistic regression model, adjusted by disease duration and age at MRI. *p* < 0.05 is marked in bold.

**Figure 3:**
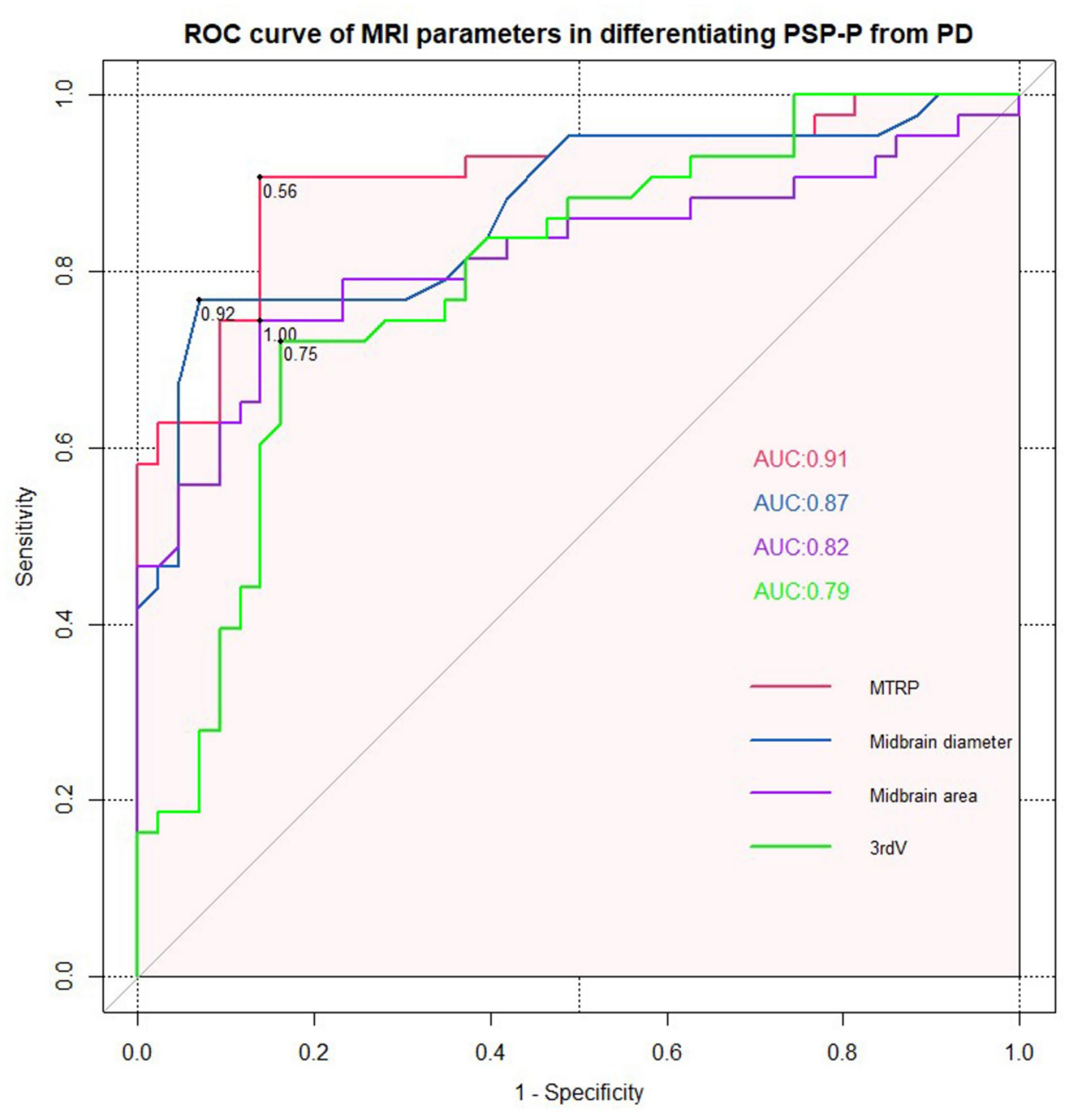
ROC curve for midbrain to pons ratio, midbrain diameter, midbrain area, and third ventricle width (PSP-P vs. PD).

Receiver operating characteristic (ROC) analyses confirmed the excellent accuracy of MTPR ≤0.56 in distinguishing PSP-P from PD (AUC = 0.91, sensitivity = 86.00%, specificity = 90.70%). In addition, midbrain diameter ≤ 0.92, midbrain area ≤ 1.00, and third ventricle width ≤ 0.75 were also suggestive signs of PSP-P (Figure 3).

## 4. Discussion

In this study, we systematically analyzed the clinical characteristics, disease progression and MRI assessment of PSP patients in mainland China. During disease progression, PSP-RS patients were more prone to falls and vertical ocular dysfunction, whereas PSP-P patients were more likely to develop tremors. By reflecting the MRI parameters of midbrain atrophy, it was confirmed that the midbrain atrophy in the PSP-RS group was more obvious than that in the PSP-P group. Longitudinal MRI changes were more pronounced in PSP-RS than in PSP-P. In addition, a series of midbrain-based MRI assessments would identify PSP-P from PD with good diagnostic accuracy.

There were 150 PSP patients in our cohort, including 100 PSP-RS patients, 44 PSP-P patients, and 6 other vPSP patients. The PSP-RS patients accounted for 66.7% of PSP cases in our cohort, while vPSP patients were for 33.3%. Respondek et al. found that vPSPs occurred in 60–75% of PSP cases in an autopsy-confirmed cohort (Respondek et al., 2014). This value was much higher than the ratio in our cohort. A potential explanation was that the typical features of vPSP did not exist, or it would only be apparent after a few years of disease (Respondek et al., 2017). This may lead to missed diagnosis of vPSP patients. As we observed, the disease duration of PSP-P patients was obviously longer. This was consistent with previous report that patients with PSP-P and PSP-progressive gait freezing (PSP-PGF) experienced a long delay in diagnosis, because they have similar clinical trajectory and initial clinical characteristics as PD (Jabbari et al., 2020). One study demonstrated that a number of patients (9.1%) with an initial diagnosis of PD developed PSP-P during a 4-year follow-up (Quattrone et al., 2019). By contrast, PSP-P and PSP-PGF were associated with slower rates of disease progression, which may also have led to a delay (Jabbari et al., 2019). There was a significant sex difference in our PSP cohort, with more men than women. This phenomenon might be explained by differences regarding sex-based comorbidities at a higher age or the lower life expectancy of older men (Chiu et al., 2010; Jecmenica-Lukic et al., 2014). Additionally, recent studies have proposed that sex-based differences exist in PSP regarding the clinical characteristics, progression, and survival of the disease (Mahale et al., 2022).

Then, clinical features and MRI image presentations between PSP-RS group and PSP-P group were further compared. Patients in the PSP-RS and PSP-P groups mainly exhibited distinct motor symptoms throughout the disease. The former was dominated by postural instability, and the latter by parkinsonism. Previous research found that the PSP-RS phenotype had a shorter interval from disease onset to clinical milestones—such as frequent falls, severe dysphagia, speech difficulties, and wheelchair dependence—while the PSP-P phenotype had a more favorable course and longer survival (O'Sullivan et al., 2008; Jecmenica-Lukic et al., 2014; Glasmacher et al., 2017). Moreover, the tau burden was significantly higher in the PSP-RS than PSP-P (Williams et al., 2007). In short, there were differences in PSP-P and PSP-RS in terms of pathological characteristics, clinical progress and prognosis. Thus, early diagnosis and detection of PSP-P is critical.

A series of midbrain-based MRI morphometric assessments have provided clues to differentiate PSP from other parkinsonian disorders. However, there have been relatively few studies of MRI assessment of different PSP phenotypes. Picillo et al. found that the midbrain area, MBTegm, and MTPR showed significant differences between patients with PSP-RS and vPSP (Picillo et al., 2020). It was comparable to our observations that PSP-RS had a lower midbrain diameter, MTPR, midbrain area, Ma/Pa, and MBTegm than PSP-P. Moreover, MRI biomarkers may be predictors of the prognosis and disease features of PSP. The MRPI accurately predicted the appearance of vertical supranuclear gaze palsy in patients with PSP-P, and reduced midbrain area was significantly associated with greater ocular motor dysfunction, more rapid disease progression, and earlier death (Quattrone et al., 2016; Cui et al., 2020; Picillo et al., 2020). Although MRI plays an important role in supporting the clinical diagnosis of PSP, longitudinal MRI studies involving patients with PSP in the early symptomatic phase were insufficient.

In our cohort, the longitudinal MRI changes were investigated in patients with PSP. The annual rel.ΔMTPR and rel.Δ (Ma/Pa) for PSP were 5.55 and 6.52%, respectively; additionally, PSP-RS presented a higher decline rate than PSP-P. The possible reason was that PSP-P patients presented with a slower rate of progression than PSP-RS. Previous longitudinal MRI studies reported that the relative 1-year decline in MTPR in patients with PSP was 4.74%, with greater midbrain atrophy in patients with PSP than in those with PD (Hwang et al., 2017; Kannenberg et al., 2021). Midbrain-based measurements, including the MRPI and MRPI 2.0 scores, progressed differently in PSP-RS and PSP-P (Quattrone et al., 2020); The rate of midbrain atrophy changes with disease progression requires further exploration. An interesting finding was that the two MRI scanning intervals of the patient were only half a year, but midbrain-based measurements changed significantly. Previous studied found that interval of 6 months could reveal imaging atrophy and clinical progression in PSP patients (Whitwell et al., 2012; Dutt et al., 2016). In total, the clinical evaluation and MRI examination of patients with atypical parkinsonian syndrome every six months were appropriate, which could help clinicians identify PSP. Longitudinal MRI studies involving more patients with PSP were essential for the discovery of potential *in vivo* imaging markers for early diagnosis.

As mentioned above, PSP-P was difficult to diagnose in the early stages and was frequently misdiagnosed as PD initially. They had similar clinical trajectories and initial clinical features but differed in their responses to treatment and prognosis. Early diagnosis and detection of PSP-P was critical. Our result was consistent with previous studies that midbrain-based MRI parameters had significantly decreased in the PSP-P group compared with those in the PD group (Alster et al., 2020). Moreover, among them, MTPR ≤0.56, midbrain diameter ≤ 0.92, midbrain area ≤ 1.00, and third ventricle width ≤ 0.75 could identify PSP-P from PD with good diagnostic accuracy. With the development of neuroimaging research, several single photon emission computed tomography (SPECT) studies have shown that the hypoperfusion subregions of cerebellar and frontal lobe were feasible in identifying PSP-P from MSA-P (Alster et al., 2022; Madetko-Alster et al., 2022). Future studies should verify these approaches in the larger cohort.

Our study has several limitations. Firstly, Information was mainly compiled from the medical history, which may be less accurate due to the biased memory of patients; however, movement disorder specialists have reconfirmed the information through structured interviews with the patients or their caregivers. Secondly, diagnoses were made based on clinical criteria, since we could not access pathologically-proven cases in this study; however, the enrolled patients who fulfilled the clinical criteria were diagnosed with possible or probable PSP with relatively high specificity. Thirdly, despite the fact that PSP is a rare disorder, the number of participants with PSP in our study was relatively small, especially in the vPSP patients. Additionally, the number of cases with longitudinal MRI images was small, and the intervals between the different cases varied; therefore, further studies with larger cohorts, including different PSP phenotypes, are warranted to identify biomarkers that support the clinical phenotypic categorization of patients with PSP.

## 5. Conclusion

This study with a relatively large PSP cohort suggested differences in clinical presentation, MRI and longitudinal MRI changes between PSP-P and PSP-RS, as well as MRI parameters that might serve as potential imaging markers to differentiate PSP-P from PD.

## Data availability statement

The original contributions presented in the study are included in the article/supplementary material, further inquiries can be directed to the corresponding author.

## Ethics statement

The studies involving humans were approved by the Ethics Committee of Xiangya Hospital, Central South University. The studies were conducted in accordance with the local legislation and institutional requirements. The participants provided their written informed consent to participate in this study.

## Author contributions

YW and QY: conceptualization, investigation, formal analysis, data curation, and writing – original draft. BJ, WZ, YaZ, QX, HZ, LW, XL, YuZ, JW, JG, XY, HJ, and BT: investigation and formal analysis. JL: formal analysis and writing – review and editing. LS: conceptualization, investigation, and writing – review and editing. All authors contributed to the article and approved the submitted version.

## Funding

The study was supported by the National Key R&D Program of China (Nos. 2020YFC2008500, 2018YFC1312003), the National Natural Science Foundation of China (Nos. 81501110, 81671075, 81971029, 82071216, 81901171), National Major Projects in Brain Science and Brain-like Research (No. 2021ZD0201803), Hunan Innovative Province Construction Project (No. 2019SK2335), and the Youth Science Foundation of Xiangya Hospital (No. 2018Q020).

## Conflict of interest

The authors declare that the research was conducted in the absence of any commercial or financial relationships that could be construed as a potential conflict of interest.

## Publisher’s note

All claims expressed in this article are solely those of the authors and do not necessarily represent those of their affiliated organizations, or those of the publisher, the editors and the reviewers. Any product that may be evaluated in this article, or claim that may be made by its manufacturer, is not guaranteed or endorsed by the publisher.
